# Using simulation to uncover care aides physiological and emotional responses to their work: A research protocol

**DOI:** 10.1371/journal.pone.0325765

**Published:** 2025-07-17

**Authors:** Patricia Morris, Rose McCloskey, Karen Furlong, Jennifer Moore

**Affiliations:** 1 Faculty of Nursing, University of New Brunswick, Fredericton, Canada; 2 Department of Nursing and Health Sciences, University of New Brunswick, Saint John, Canada; 3 Research Services, University of New Brunswick, Fredericton, Canada; PLOS: Public Library of Science, UNITED KINGDOM OF GREAT BRITAIN AND NORTHERN IRELAND

## Abstract

Care aids play a vital role in long-term care homes by providing essential support to residents, assisting with daily activities, and ensuring that individuals receive the care and attention they need. These professionals are often the primary point of contact for residents, making their well-being a crucial aspect of long-term care. However, the demanding nature of the job can lead to high levels of stress, and job dissatisfaction. While much is known about the antecedents to stress and subjective experiences of job-stress in LTC, there has been limited research to quantify the mental workload of that work. This paper describes the protocol for our research project that will use simulation to measure the mental workload of care aides while they conduct routine care to residents. Specifically, study participants will take part in simulation involving caring for a resident with dementia, which entails common challenges encountered by care aides including time pressure and resistance to care. Mental workload will be measured using physiological responses including heart rate, heart rate variability, and pupil diameter. This research promises to extend existing knowledge on care aides’ experiences and provide a deeper, multidimensional understanding of stress and mental well-being in caregiving roles. This innovative approach will not only validate qualitative insights but also uncover new dimensions of care aides’ work that may have been overlooked, paving the way for more targeted interventions and support strategies in the workplace.

## Introduction

Working in long-term care (LTC) can be physically and psychologically demanding [1,2]. Staff in LTC are primarily unregulated care aides who are predominantly women with little, if any, formal qualifications [3]. They often have limited access to continuing education [4]. Despite providing most of the hands-on care to residents, care aides are not always viewed as essential members of the care team. Research reveals that care aides are often marginalized in the workplace [5,6]. Their care work is demanding and complex, and yet they have very few opportunities and limited autonomy to make decisions pertaining to the care they provide [3]. Banerjee and colleagues [5] describe the work of care aides as occurring in highly regimented environments, where they are expected to follow strict routines and often struggle to “balance the tension between bureaucratic requirements and the immediate, individual needs of elderly residents” (pg. 29). Under such conditions, care aides report high levels of workplace stress [4,7,8] and emotional exhaustion [9,10].

Care aides attend to people with complex health and social needs, including high levels of chronic health conditions, physical disabilities, and cognitive impairments [11]. Despite an increase in the complexity of residents’ profiles over recent years, staffing levels have remained largely unchanged to meet the increased demand for care [12,13]. It is under these increasingly dire conditions that care aides are reported to have a maximum of 3 uninterrupted minutes to complete many of their care tasks [14], often leading to rushed or missed care [15]. This care is provided predominantly (more than 80%) by care aides [16]. There are reports of a prevailing LTC culture characterized by rigid policies, prescriptive practices, and hierarchical labour conditions. Care aides often have little or no autonomy in their work [11,17,18]. This can lead to significant challenges when interacting with residents’ families, whose expectations of care often exceed what is feasible within the organizational constraints [19,20]. There is widespread concern that the highly regulated nature of LTC does not support quality, person-centered care and that time pressures direct care aides to react and respond to residents in routinized ways [21,22]. Canadian researchers report that prescriptive LTC regulations impact frontline workers’ abilities to engage in teamwork, integrate health and social care, and provide compassionate and supportive care [21,23].

Two recent systematic reviews report high levels of job-related stress among those working in LTC [24,25]. Antecedents to job-related stress in care aides include contextual factors such as time pressures [26], low social capital [24], and unrealistic work demands [27–30]. According to Statistics Canada’s most recent analysis, a heavy workload is the most common predictor of employees’ levels of job-related stress [31]. Workers employed in health and social care were more likely than the average worker to describe a heavy mental workload (32.3%, compared with 23.7% on average) and a heavy emotional load (21.4%, compared with 11.7% on average) in their work. While much is known about the antecedents to stress and people’s subjective experiences of job-stress in LTC, there has been limited research to quantify the mental workload of that work. Care aides’ physiological responses to the physical and emotional demands of their work are an important factor to consider in quantifying their mental workload in LTC environments. Understanding how care is enacted in practice and staffs’ physiological responses to work-related events is necessary to address the conditions of care aides’ work. Measures of mental workload and the impact of everyday care practices in LTC could potentially play a significant role in identifying when and where measures can be introduced to support care staff and ultimately facilitate quality resident care.

This protocol for research into mental workload via physiological measures leverages simulation as a research methodology. The LTC environment presents unique challenges for researchers attempting to study the work conditions of care aides. For instance, residents are typically frail and observing their care presents complex ethical challenges that researchers must overcome, such as fluctuating capacity to provide consent [32]. Resident safety can also be easily compromised by the presence of an outside observer, and introducing data collection measures can alter staff-client interactions. By reproducing real-world phenomenon, simulation allows for the exploration of questions that are otherwise inaccessible in a clinical setting [33]. A high degree of validity can be established in simulated research environments that reflect the clinical setting.

### Theoretical framing

Mental workload is a factor in human performance that affects mental processing of information and work performance. As a multidimensional construct, mental workload reflects the accumulation of different work-related demands and is the product of interactions between task requirements, circumstances in which a task is performed, and one’s perception of the task [34,35]. Mental workload can be modified with the appropriate environmental, regulatory and/or individual supports [36]. Optimizing mental workload can positively impact work performance, mitigate errors and improve job satisfaction [35,37]. When mental workload is low (i.e., prescriptive work), workers can become frustrated and disengaged; however when it is high (i.e., complex residents) staff can be overwhelmed, their cognitive resources depleted, and they can exhibit a decrement in their performance [37,38]. The study of mental workload serves three primary purposes: i. to quantify the transactions between care aides and their work demands, and ii. to predict human performance in different situations, specifically the resource depletion and effort required of aides to perform their duties under specific circumstances, and iii. to identify needed modifications to the work environment [34,38].

### Study purpose

This study will examine mental workload of care aides during a controlled time-pressured encounter staff in LTC with a resident who resists the progression of care. Resistance to care, or refusal to advance the care encounter, occurs regularly in LTC. This is particularly common among residents with a diagnosis of dementia, and is reported to be a major source of moral distress for staff [39]. Resistance to care is exhibited in many ways, such as verbally saying no, moving away, yelling, or physically striking out [40,41]. Resisting the progression of care often means that vital care, such as baths and dressing, are not accomplished in a timely manner, if at all. Care that cannot be progressed is a significant source of stress for care aides because it can give the impression that a care provider did not fulfill their responsibilities and that the resident has been neglected [42]. In this study we aim to measure care aides’ pupil dilation and heart rate variability as proxies for mental workload when care progression is resisted by a resident, and to examine the impact of introducing external time pressures into the scenario. The study’s objectives are to i. explore the relationship between changes in biomarkers (pupil and heart rate) and individual reports of demanding work activities; and ii. explore the ability of biomarkers (changes in pupil size and heart rate variability) to differentiate between low, medium, and high demanding work activities of care aides; and iii. identify individual characteristics that influence physiological responses to work activities.

## Design and methods

This study will be a mixed-methods study that aims to collect qualitative data about participants’ experiences of working with residents who resist care in addition to physiological measurements as proxies for mental workload. In the quantitative section of the study, we will employ an exploratory cross-sectional observational study that relies on repeat measures from each participant. We will be seeking to identify the mental workload of care aides attempting to perform routine care with a resident who forestalls care progression. Care aides will take part in a standardized simulation involving caring for a resident with cognitive impairment who resists their attempts to progress the care encounter. Each participant will go through a simulation individually. Data collection is expected to take approximately 60 minutes and will include: i. pre-simulation introduction, consent, and questionnaires; ii. application of data collection equipment to obtain physiological measures; iii) a control phase; iv) baseline data collection; v) engagement in the simulation; and v) a qualitative interview about the experience and similar work experiences.

The script for the simulation was carefully crafted to produce a simulation with a high degree of fidelity. Fidelity in simulation is “the degree of realism created through the selection of simulation equipment, setting, and scenario” [43]. There are three types of fidelity: conceptual, psychological, and physical. Conceptual fidelity is the degree to which the simulated problem or situation mirrors situations that occur in the clinical context being simulated. In this study, a high level of conceptual fidelity will be achieved because we obtained feedback on the simulation script from experts in the clinical setting [44,45], including experts in long-term care, dementia, and simulation. Psychological fidelity is the degree to which participants feel the simulation captures a real-life scenario [43]. Psychological fidelity will be achieved by hiring an actress to play the role of a resident with cognitive impairment, and others to play the role of LTC supervisors. We have trialed our planned simulation and elicited feedback from individuals who work in long-term care; small refinements to our scenario were made based on their feedback. Physical fidelity refers to the “level of realism provided by the physical elements of the simulation” (Harris et al., 2020) and will be achieved because this research will be conducted in a LTC simulation lab that is designed to the exact specifications of a LTC home. The lab mirrors a LTC bedroom and bathroom, complete with mechanical lifts, care plans, and a supply room (see S1, S2 and S3 Figs for photos).

The simulation will be validated through a combination of methods that assess both the accuracy of the simulation and the performance of the actor. The feedback received from subject matter experts (previously described) was used to refine the scenario and its authenticity, ensuring that it accurately reflects care aides’ real-world situations. The actor will receive considerable training prior to the study, including orientation to simulation in general, the research specifically, and the role to be portrayed. A consistent actor is hired for the study and simulation “run-throughs” or rehearsals have been done prior to data collection. The actor has been provided feedback on their rehearsal performances by non-participant LTC staff and members of the research team with a focus on consistency, realism, and authenticity. When the study commences, participants will take part in a debriefing session after each simulation where they will be asked to provide feedback on how effective the simulation reflected real-world caregiving activities. During the study’s pilot, this feedback will be used to refine the simulation to enhance its realism and authenticity.

A pilot test of the simulation has been conducted which resulted in the refinement of the original research design. The pilot demonstrated insufficient time planned to calibrate baseline heart rate. This finding resulted in the addition of a baseline phase where participants will be asked to perform a menial activity with low physical and mental workload demands (gather supplies from a cart). The pilot demonstrated some variability across participants in the time spend in specific phase of the simulation. To ensure consistency, a timed distraction was built into each simulation to trigger the actor to progress to the next phase (as described below). The pilot also highlighted the need to adjust how the computer used for the pupillary data was carried by participants. The initial plan for participants to wear a backpack with a laptop connected to the goggles via a USB cord proved problematic, as the laptop overheated inside the bag. To address this issue, we sourced a laptop harness that could be comfortably worn like a backpack and provided adequate ventilation, preventing the laptop from overheating during the simulation.

The simulation centers on a resident living with dementia who stalls the progression of care in three progressively escalated interventional phases, with accompanying external time pressures. The study will begin with a control phase and a baseline data collection phase. Both phases will be included to establish a foundation for comparison, isolating the physiological responses associated with rest (control) and low mental workload (baseline) from those observed during the progressively escalated interventional phases

**Control phase.** During the control phase participants will sit for two minutes without gross motor movement and without reading material. During this period their heart rate variability will be measured using the attached sensors (see Measures section for specifics). This phase provides data about the participant’s heart rate variability and pupil dilation in a period of rest, to serve as a comparison for data obtained during phases 1–3 of the study. Following the control phase, care aides will be provided with the resident’s care plan and left alone to review for 3 minutes. After this period they will be escorted to a storage room set up to simulate a clean supply room by an actor playing a Registered Nurse (RN).

**Baseline phase.** During the baseline phase, participants will be asked to perform a primarily physical task with a low mental workload profile in the supply room. Namely, they will be asked to load towels and facecloths onto a cart to bring with them into the resident’s room. This baseline period will allow us to capture normal variations in the participants’ heart during movement associated with low mental workload.

**Calibration phase.** Once outside of the resident’s room, the participant will be led through a series of movements as part of a simulated stretching program. These movements include exercises that emphasize shifting weight from side to side, moving weight forward and backward, and crouching down. These movements provide an opportunity to calibrate the pupil dilation monitors in a room that has been calibrated to emit the same number of lumens as the resident’s room, ensuring consistent brightness throughout the space.

**Phase 1.** Once stretches are complete, the actor playing the RN will point to the resident’s door and instruct the care aide that the resident is sitting in her chair beside the bed. The simulated resident immediately begins forestalling care by attempting to engage the participant in other activities (e.g., coloring and talking about her special interest in horses). This phase lasts 2 minutes to ensure a sufficient sample.

**Phase 2.** An external time pressure will be applied in the form of the RN actor knocking on the door and checking in on progress. The RN will emphasize to the care aide that the resident’s family is on their way into the home and that the resident must look and smell good by the time they arrive. During this period, the simulated resident will continue to resist and will verbally escalate their resistance by talking negatively about staff. This phase lasts 2 minutes to ensure a sufficient sample.

**Phase 3.** The resident will concede to care if it is done in their bed and move toward the bed to progress the care encounter toward hands-on care provision. Once in the bed, the resident will become increasingly verbally escalated, shake her cane, and thrown a soft object (a teddy bear) at the care aide. This phase will last 2 minutes to ensure a sufficient sample.

**Phase 4.** An external time pressure will be applied when the RN returns to find the resident continues to be dressed in their night clothes, lying in bed. The RN will criticize the care aide and note that the family is very difficult to interact with and has higher expectations of staff than other families. The care aide will be instructed to hold the resident’s hands while the RN performs the care work. The simulation will end after two minutes in this phase, to ensure a sufficient sample.

### Sampling and recruitment

This study will rely on a convenience sample of care aides who work in LTC and are able to travel to the simulation lab where the study will take place. To be eligible, participants must be able to understand and converse with a simulated resident in English and have worked a minimum of 3 months in a LTC home on a part- or full-time basis. No limits will be placed on age, gender, race, or other demographic variables. Nursing students working as care aides while attending school will be ineligible as their role is only temporary and it is anticipated that they will fulfill roles with more social capital in the foreseeable future. Care aides who are on orientation in a new role will be excluded from the study, as their limited experience in the position may not provide a reliable representation of standard caregiving practices.

We aim to recruit a diverse sample that reflects the local context by ensuring participants represent a range of ages and experiences. For example, the study will actively recruit both younger nurses with less than five years of experience and seasoned professionals with over 20 years in the field. Additionally, the protocol will seek participants from various LTC homes in the area, ensuring a mix of small (less than 60 beds), medium (60–119 beds), and large (120 of more beds) to capture different caregiving environments. This approach will help ensure that the findings are applicable to a broad spectrum of care providers within the local community. The goal is to recruit a minimum of 24 participants, based on a power analysis specific to these repeated measures study.

Recruitment will involve emailing licensed, publicly funded LTC homes in the region where the study is being conducted. An individualized email will be sent to care home administrators explaining the nature and purpose of the study, along with a request to display a recruitment poster in areas where they will be visible to staff (i.e., staff rooms). We will also employ a snowball technique by asking staff who contact us to share information about the study to their networks. A $50 VISA gift card will be given to participants as a token of appreciation.

### Measures

Prior to the simulation, participants will be asked to complete a demographic survey which will include age, gender, education level, and relevant background information such as experience in LTC. Participants will also complete the Strain in Dementia Care Survey (SDCS) and the Dementia Policy Questionnaire (DemPol-Q). The SDCS consists of 27 items pertaining to perceived level of job strain among care staff [46]. Data collected from the SDCS is on a four-point Likert Scale with a higher score indicating a high level of job strain. The DemPol-Q consists of 19 dichotomized items about the existence and helpfulness of institutional policies in the delivery of resident care [47] A modified version of the DemPol-Q will be used to reflect the scope of practice of care aides within the region where the study will take place. Specifically, only 6 of the 19 items in the questionnaire will be used. This modified version of the scale has an acceptable internal reliability (Cronbach alpha = 0.734).

During the simulation, physiological data will be collected using portable heart rate monitors and goggles that measure pupil direction and dilation. Heart rate and heart rate variability will be measured using Firstbeat Bodyguard© that involves a 2-lead continuous heart rate monitoring (Firstbeat Technologies Ltd, Jyväskylä, Finland). Heart rate and heart rate are widely used physiological measure to assess stress and mental workload [48]. These measures reflect the activity of the autonomic nervous system, which regulates involuntary functions such as heart activity.

Heart rate and heart rate variability are processed inside of the Firstbeat Bodygaurd© device and the data is then exported via a USB port. The Firstbeat Bodygaurd© has been previously tested in both clinical and laboratory investigations and found to be a valid and reliable measure of heart rate and heart rate variability [49,50].

Peabody and colleagues also reported no significant difference in data collected via the Firstbeat Bodyguard© during simulations and actual clinical activities [51]. Application of the Firstbeat Bodyguard© will be in compliance of established guidelines put forth by Quigley et al. [52] including the modified Lead II (torso) placement of electrodes, focus on changes in data from baseline, two-minute intervals between stimulus and change score measures, and the standardization of stimuli and recording both within and between study comparisons.

Pupil diameter will be continuously measured during the simulations using Pupil Lab© googles (Pupil Labs GmbH, Berlin, Germany) and software. The googles and software consist of a 3D printed eyeglass frame with built in cameras that record pupil size and movements. The open-source Pupil Lab © software is installed on a lab computer dedicated specifically for this study, with the googles connected to the computer through a USB cable. These goggles feature two infrared cameras directed at each eye and a wide-angle world-view (front-facing) camera. The eye cameras capture 192 px images sampled at 200 Hz with 0.60° accuracy (after calibration) and account for slippage of the goggles using 3D models of pupil diameter. Pupillary activity, specifically changes in pupil size and dynamics, are widely recognized as physiological indicators of mental workload and stress [53–55], particularly when used in conjunction with cardiovascular measures such as heart rate [56]. Given the sensitivity of pupil responses to light conditions, consistency in lighting will be assured during the calibration phase of the study. The goggles will be fitted to participants by adjusting the length of the camera arms and will be calibrated initially by asking participants to hold their eyes on a fixed point on the wall while moving their head in the largest range of motion possible without moving their eyes. Calibration will be confirmed when the camera feed displays a clear image of the pupil and an estimate of its size. Baseline measurements of pupil size will be obtained, and consistent sources of light used across all simulations to control external stimuli and accurately measure how pupils respond to specific conditions. Participants will be required to wear these non-invasive googles throughout the simulation to allow for continuous measures of pupils. If necessary, these goggles can be worn in conjunction with eye glasses. The validity and reliability of Pupil Labs© goggles have been established through several studies and evaluations [57,58].

All simulations will adhere to a standard protocol to ensure consistency across all research conditions. This includes maintaining uniform experimental design, using calibrated equipment, and following predetermined procedures for participant interaction. The actor will adhere to a strict protocol that includes maintaining precise periods of time in each of the previously described four stages of the simulation. By standardizing these elements, we can control extraneous variables, minimize errors, and ensure that each participant has an equal and consistent experience. This consistency is vital for obtaining reliable data, enabling accurate comparisons, and enhancing the validity of the study. Standardization also facilitates the [57] reproducibility of the research, allowing other researchers to replicate the study under the same conditions and verify the results.

After the simulation, semi-structured one-on-one interviews will be conducted with participants. The focus of these interviews will be twofold: first, to gather feedback on how accurately the simulation reflects an encounter with a resident, and second, to obtain insights on the experience of working under the conditions created in the simulation, namely, time pressure, resistance to care by residents, meeting family expectations, and conforming to institutional expectations. Specific questions will include how did this simulation compare to working with a resident who resists care?; tell me about a time that you found it challenging to care for a resident and what made this situation particularly challenging; what aspects of residents’ resistance to care do you find the most difficult?; How does residents’ resistance to care impact your ability to fulfill your responsibilities at work?; How stressful do you find caring for residents who resist care, and what factors contribute most to the difficulty of these situations?

### Ethics

Ethical approval for this protocol was obtained from the Research Ethics Boards of the academic institution of the investigators (File #2024-064). A member of our team will review a standardized consent form with potential participants, emphasizing the voluntary and confidential nature of the study. Participants will be informed about the video recording of the simulation, and if uncomfortable with the recording, they can have their activities manually documented by an observer. The privacy and security measures in place for the recording software, and the option to participate without being recorded will be emphasized. A member of our research team will review the consent form with potential participants. Written consent will be obtained from participants who will also be provided a copy of the consent form for their own records, and informed that they can stop simulation at any point or refrain from answering any questions. Those who stop the simulation of withdraw from the study will still qualify for the $50 gift card.

The study will use an actor to simulate a resident with a cognitive impairment. In addition to creating more realistic and standardized scenarios than a mannequin/simulator could create, there are several ethical benefits of using an actor over a real person with cognitive impairment. Using an actor for the study avoids placing individuals with cognitive impairment in unnecessary and reasonable stressful situations. Actors can also be fully informed of the nature and purpose of the study, which may not be feasible when working with individuals who have cognitive impairments. The study’s focus on staff responses to distressed residents who resist care is more effectively achieved with an actor, as this approach enables the scenario to be consistently recreated across participants within a standardized timeframe [59,60].

### Data management and security

All data and the results will be kept indefinitely on a password protected secure server and will only be accessible to members of the research team. Recordings of simulations will be deleted immediately after the required data is extracted. SimCapture software has data security and safety certification. The simulation software also underwent a rigorous data security and privacy assessment from our institutional privacy officer.

### Data analysis

Data will be examined individually and in combination to identify and assess associations and interactions between variables and outcomes. Analysis of pre-simulation data will be conducted to describe and compare the sample by age, experience, and education level. Data from the simulations will be paired with the continuous data collected via Firstbeat BodyGaurd© and Pupil Labs ©. Data of interest is the changes from baseline in heart rate and pupillary sizes collected during the baseline and calibration phases previously described. Recorded simulation data to be analyzed will be the precise moment and duration of the planned stressor within in the simulation (e.g., time pressure, resistance to care, organizational and family expectations). Combining video recorded data with physiological data allows for a synchronized, minute-by-minute profile of participants’ autonomic responses to events within the simulation. To account for individual physiological differences, changes from each participant’s baseline heart rate activity and pupil diameter will be used to calculate responses to stressors. Regression analysis and statistical modeling techniques will be employed in an attempt to understand the role of workplace strain (SDCS) and perceived involvement in planning care and quality improvement (DemPol-Q) within LTC where participants work.

Post-simulation qualitative data will be analyzed using Clarke and Bruan’s recursive six-phase inductive process for thematic analysis [61]. This process will involve becoming familiar with the interview data, identifying themes and patterns across data, defining themes, and reporting findings. To ensure rigor and transparency, presuppositions will be identified prior to initiating analysis of data and a continuous process of reflexivity will be used throughout. Results of this analysis will provide important information on the feasibility of simulation to understand mental workload of care aides, and to determine comparability of physiological and qualitative data.

## Timeline

Recruitment of the participants began in late fall 2024. All participants have been recruited, and simulations are expected to be completed by May 2025. The data analysis will be conducted by September 2025 and the manuscript will be completed by December 2025.

## Conclusion

Despite their essential role, care aides have been largely overlooked in healthcare research [62]. This lack of attention not only undermines their contributions, but also limits how we can help improve their working conditions, address their unique challenges, and ensure their well-being. Without a clear understanding of the impact of the work on their well-being, it is difficult to develop effective practices to support them and it is challenging to provide adequate training. This oversight perpetuates issues such as burnout, high turnover rates, and insufficient recognition, ultimately impacting the quality of care they provide to residents.

This study will produce high-quality, rigorous data on the physiological impact of providing care for residents in LTC under time pressures. To our knowledge, this is the first study that will examine the physiological responses of care aides to their everyday work demands, albeit during a simulation. This research promises to extend existing knowledge on care aides’ experiences and provide a deeper, multidimensional understanding of stress and mental well-being in caregiving roles. This innovative approach will not only validate qualitative insights but also uncover new dimensions of care aides’ work that may have been overlooked, paving the way for more targeted interventions and support strategies in the workplace. This research also has the potential to examine aspects of LTC that have traditionally been unattainable. Simulation offers an opportunity to re-create the conditions in which care aides work. In the context of this research, simulation will allow a clinical investigation to be conducted without entering LTC. It will enable our team to explore care aides’ responses to potentially stressful encounters in a safe way that can be terminated at any point without disrupting resident care. It can also be done without unnecessarily exposing residents to potential harm from time-pressured care for research purposes. Simulation will allow researchers to continue their efforts by reproducing real-world phenomena to answer clinical research questions. Simulation offers the potential to transform how clinical research is conducted in LTC, including how new knowledge is generated and tested.

## Supporting information

S1 Fig Photo of a resident bedroom in the simulation lab. (DOCX)

S2 Fig Photo of the resident bathroom in the simulation lab. (DOCX)

S3 Fig Photo of the resident tub room in the simulation lab. (DOCX)
