# Supplementary figures and images for: Using simulation to uncover care aides physiological and emotional responses to their work: A research protocol
Source: PLoS One. 2025 Jul 17;20(7):e0325765. doi: 10.1371/journal.pone.0325765 (PMC12270176; doi:10.1371/journal.pone.0325765)

S1 Photos of Simulation Lab


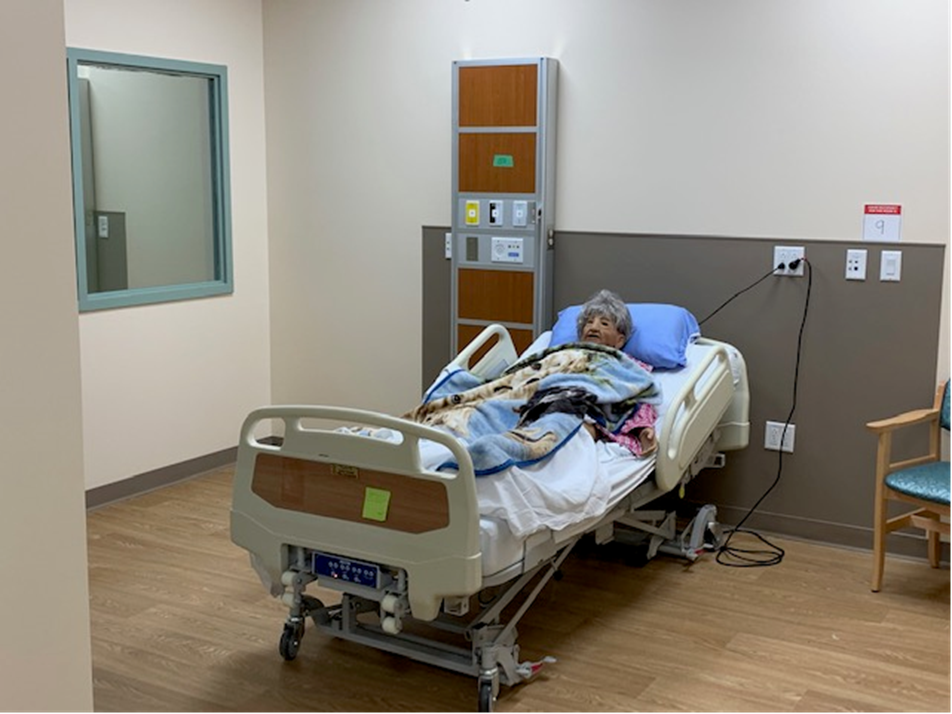


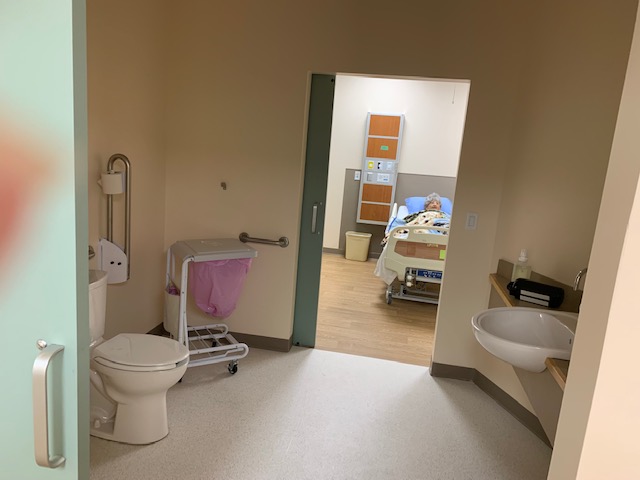


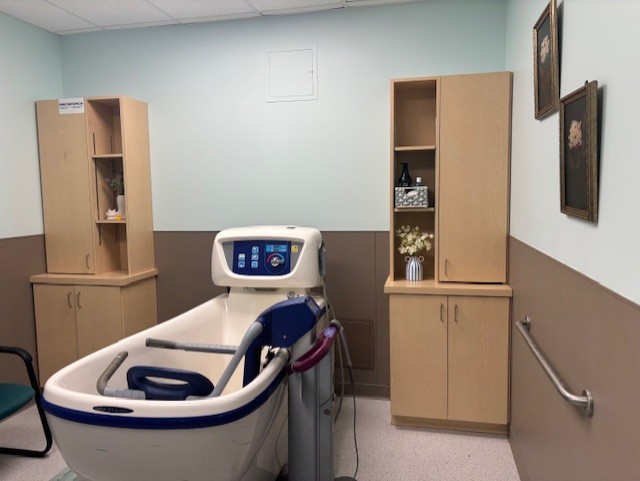

Supplement: S1 Fig — (DOCX) [file pone.0325765.s001.docx]

S2 Figure Photo of Bedroom and Bathroom Simulation Lab


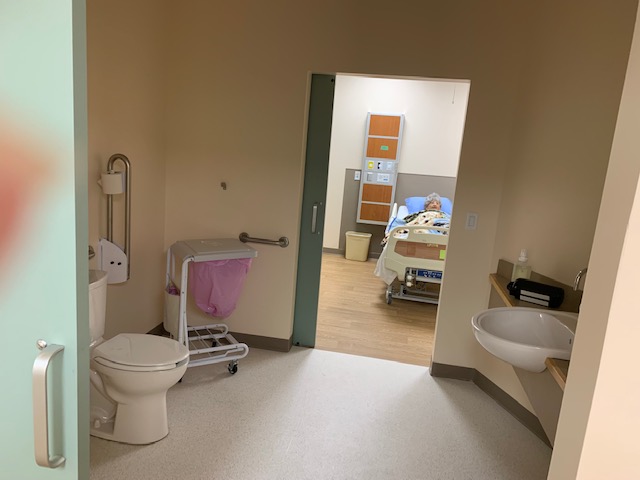

Supplement: S2 Fig — (DOCX) [file pone.0325765.s002.docx]

S3 Figure Photo of Tub Room in Simulation Lab


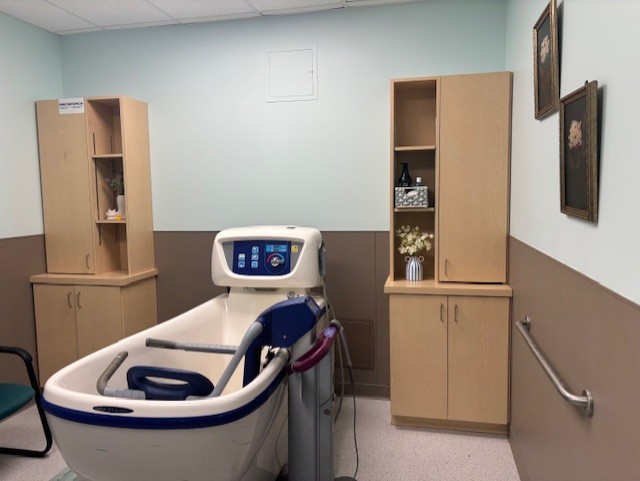

Supplement: S3 Fig — (DOCX) [file pone.0325765.s003.docx]
